# Supplementary material for: Gait Analysis of Bilateral Knee Osteoarthritis and Its Correlation with Western Ontario and McMaster University Osteoarthritis Index Assessment
Source: Medicina (Kaunas). 2022 Oct 9;58(10):1419. doi: 10.3390/medicina58101419 (PMC9610794; doi:10.3390/medicina58101419)
Supplement: Supplementary file 1 [file medicina-58-01419-s001.zip › Supplementary Material.pdf]

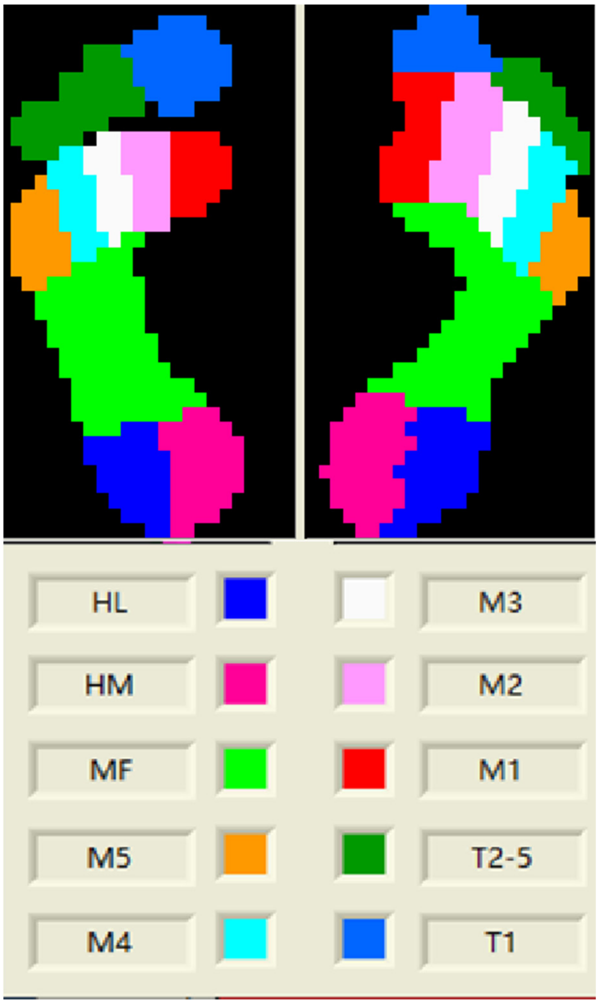

The gait analysis instrument used in this experiment divided the sole of the foot into 10 separate areas including: Heel Lateral (HL), Heel Medial (HM), Mid Foot (MF), Metatarsal 1 (M1), Metatarsal 2 (M2), Metatarsal 3 (M3), Metatarsal 4 (M4), Metatarsal 5 (M5), Toe 1 (T1), and Toe 2-5 (T2-5). The pressure in each area was represented by a different colored curve. The "m" and "n" shape pressure pink curves of Figure 2 represent the sum of 10 separate areas of the sole of the foot.
