# Supplementary figures and images for: Gait Analysis of Bilateral Knee Osteoarthritis and Its Correlation with Western Ontario and McMaster University Osteoarthritis Index Assessment
Source: Medicina (Kaunas). 2022 Oct 9;58(10):1419. doi: 10.3390/medicina58101419 (PMC9610794; doi:10.3390/medicina58101419)

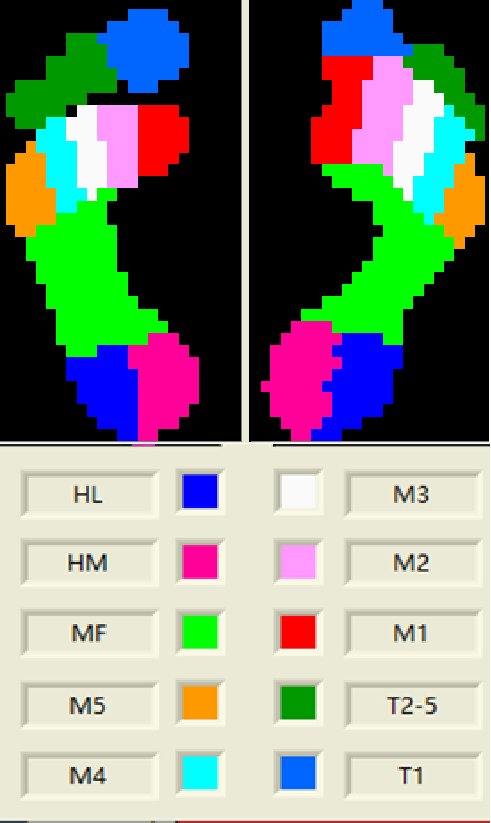

Supplement: Supplementary file 1 [file medicina-58-01419-s001.zip › Supplementary figure.tif]
